# A Case of Multisystem Inflammatory Syndrome Due to SARS-CoV-2 Presenting With Acute Appendicitis Symptoms

**DOI:** 10.7759/cureus.22206

**Published:** 2022-02-14

**Authors:** Cüneyt Uğur, Uğur C Mete, Ethem Ömeroğlu

**Affiliations:** 1 Department of Pediatrics, University of Health Sciences, Konya City Health Application and Research Center, Konya, TUR; 2 Department of Pediatrics, Konya City Hospital, Konya, TUR; 3 Department of Pathology, Konya City Hospital, Konya, TUR

**Keywords:** gastrointestinal symptoms, acute appendicitis, child, mis-c, sars-cov-2

## Abstract

Multisystem Inflammatory Syndrome (MIS-C) in children associated with SARS-CoV-2 infection has a variable clinical presentation because it affects many systems. It can affect the cardiac, renal, respiratory, hematological, gastrointestinal, dermatological, and neurological systems. If left untreated, it causes fatal complications. In this case report, a five-year-old male patient was admitted to the pediatric emergency service with complaints of fever, nausea, vomiting, abdominal pain, and loss of appetite. Physical examination revealed tenderness, defense, and rebound in the right lower quadrant of the abdomen. On ultrasound, the diameter of the appendix was determined as 6.8 mm. The patient, who was operated on for acute appendicitis (AA), was re-evaluated after fever and vomiting did not resolve, and he was diagnosed with MIS-C. This case was presented to remind that MIS-C should be excluded before the diagnosis of AA in patients with fever for more than 24 hours, gastrointestinal symptoms, and findings of AA.

## Introduction

Multisystem Inflammatory Syndrome (MIS-C) in children associated with SARS-CoV-2 infection has a variable clinical presentation because it affects many systems. The findings appear weeks after infection with SARS-CoV-2 [1]. MIS-C diagnostic criteria have been determined by WHO and the Centers for Disease Control and Prevention (CDC) [2]. Fever >38℃ for more than 24 hours, involvement of at least two systems [cardiac, renal, respiratory, hematological, gastrointestinal (GI), dermatological, or neurological], exclusion of other possible alternative diagnoses, elevated inflammatory markers, and positive reverse transcription real-time polymerase chain reaction (PCR), antigen test, or serology; or any contact with patients with COVID-19, the diagnosis of MIS-C is made [2,3]. This case was presented to remind that MIS-C should be excluded in patients with fever for more than 24 hours and GI symptoms and suspected acute appendicitis (AA).

## Case presentation

A five-year-old male patient was admitted to the pediatric emergency service with complaints of fever, nausea, vomiting, abdominal pain, and loss of appetite. In the physical examination, he was conscious his general condition was poor, slightly dehydrated, the oropharynx was hyperemic, and tenderness, defense, and rebound were detected in the right lower quadrant of the abdomen. There was no rash, and other system examinations were normal.

**Figure 1:**
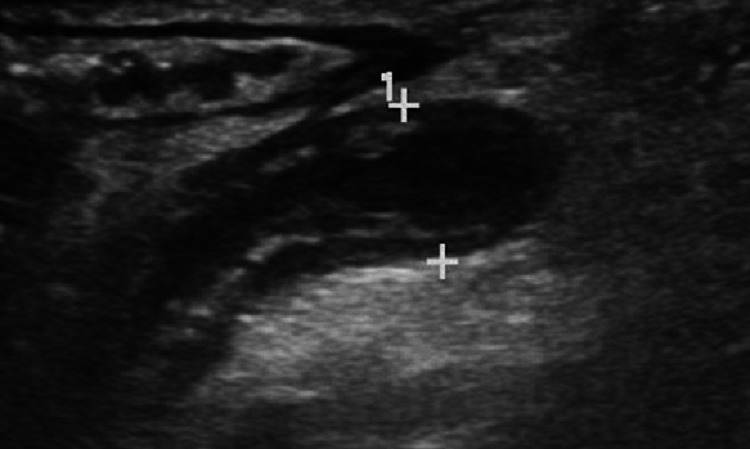
Ultrasonography image of the appendix and surrounding tissues.

In the laboratory tests; white blood cell (WBC) 18.03 103/μL (neutrophil 13.8 103/μL, lymphocyte 2.15 103/μL), hemoglobin 12.6 g/dL, platelet 363 103/μL, C-reactive protein (CRP) 58,8 mg/L, glucose 70 mg/dL, blood urea nitrogen (BUN) 11 mg/dL, creatinine 0,39 mg/dL, aspartate aminotransferase (AST) 26 U/L, alanine aminotransferase (ALT) 8 U/L, sodium 134 mmol/L, potassium was 4.8 mmol/L. Urine examination was normal. Abdominal ultrasonography (USG) was performed on the patient suspected AA with these clinical and laboratory findings. Abdominal USG performed at the time of admission was reported as “…The mesentery wall is thick and edematous. The diameter of the appendix is 6.8 mm, and its wall is edematous. Fluid was observed around the appendix.’’. The USG image of the appendix and surrounding tissues is shown in Figure 1.

With these findings, the patient was admitted to the ward with the diagnosis of AA by the pediatric surgeon and was operated on afterward. The patient, whose complaints of fever and vomiting continued on the first postoperative day and diarrhea began on the second day, was consulted by a pediatrician.

In the physical examination performed by the pediatrician, consciousness was clear general condition was moderate and frail. In the abdominal examination, bowel sounds were increased, and there was an operation scar in the right lower quadrant. There was widespread tenderness in the abdomen, and there weren’t defenses and rebound. Other system examinations were normal. After the patient was evaluated, necessary tests were requested with the suspicion of MIS-C.

**Figure 2:**
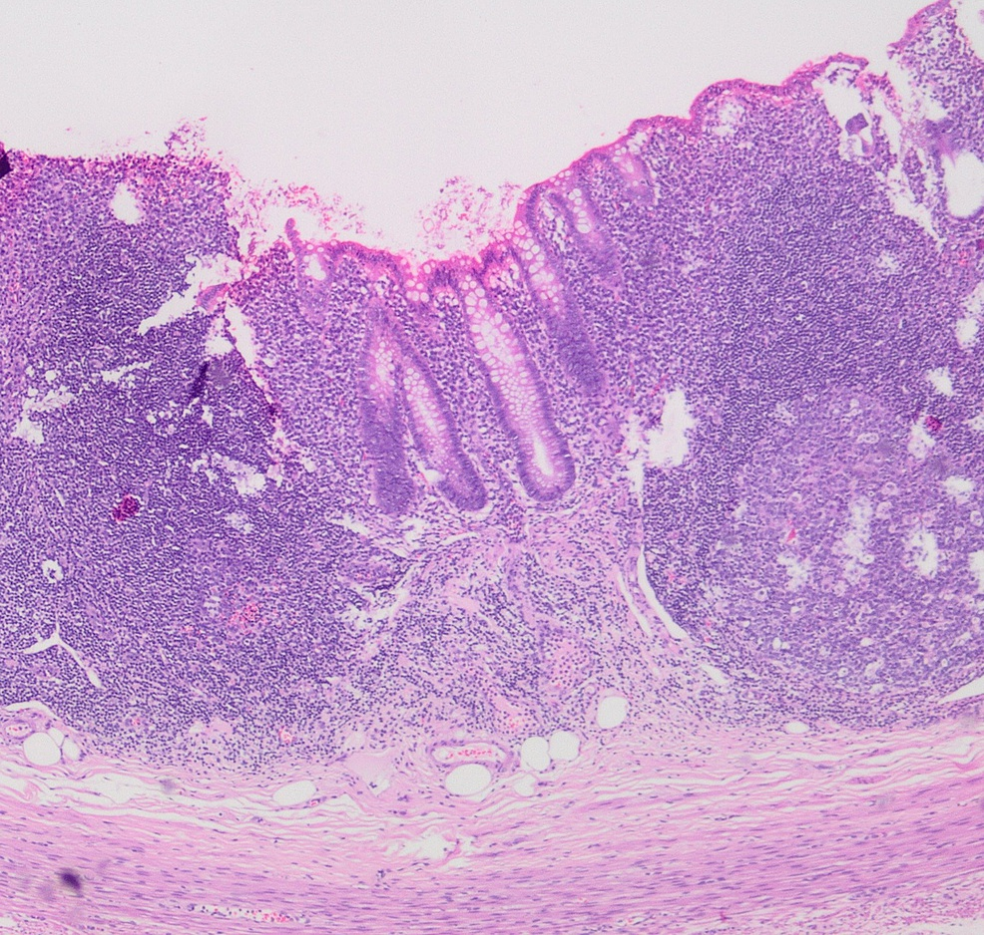
In the appendix, intact mucosa, lymphoid tissue with the enlarged germinal center, lymphoid hyperplasia, and lymphocyte infiltration without neutrophils in the muscular layer.

**Figure 3:**
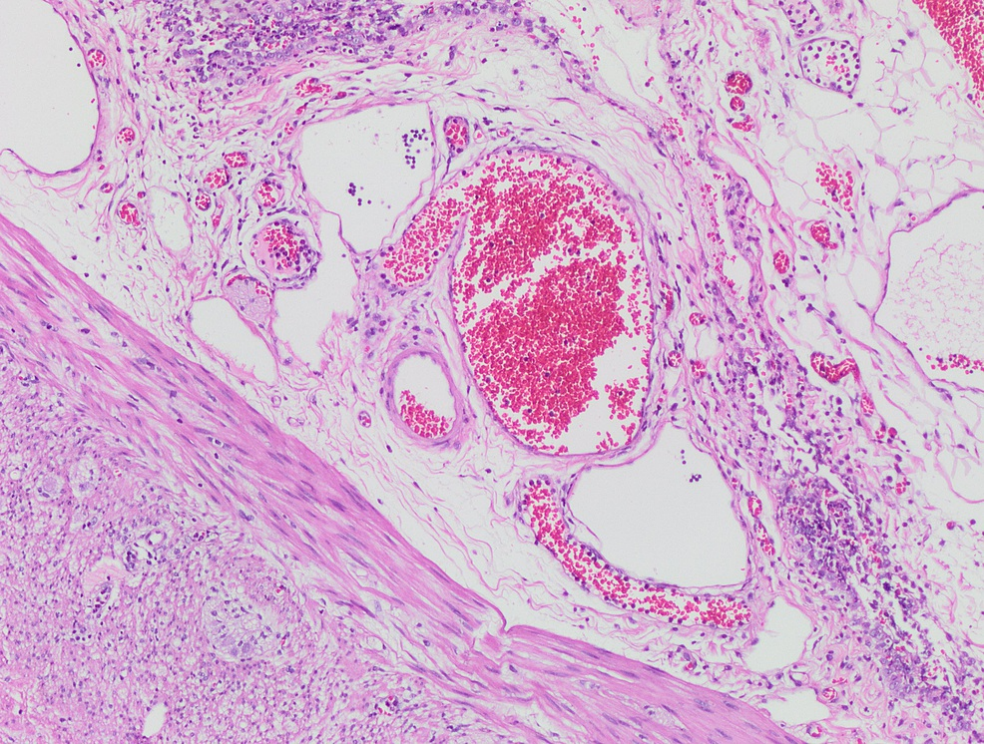
In the mesoappendix, lymphocyte and histiocyte infiltration around the enlarged congested vascular structures.

In the patient's laboratory tests; WBC 10.24 103/μL (neutrophil 6.76 103/μL, lymphocyte 2.33 103/μL), hemoglobin 11.3 g/dL, platelet 344 103/μL, CRP 81.4 mg/L, sedimentation 51 mm/h, procalcitonin 3.13 μg/ L, ferritin 147 μg/L, fibrinogen 7.32 g/L, D-dimer 17.38 mg/L, prothrombin time 12.8 sec, international normalized rate 1.13, active partial thromboplastin time 23.4 sec, troponin 4.5 ng/L, N-terminal pro B- type natriuretic peptide 87.77 ng/L, creatinine 0.29 mg/dL, BUN 6 mg/dL, AST 24 U/L, ALT 8 U/L, albumin 40 g/L, sodium 138 mmol/L, potassium 3.8 mmol/L, calcium was 9.7 mg/dL. The patient's COVID-19 PCR result was negative, and the COVID-19 IgG antibody result was positive. Adenovirus and rotavirus were not detected in the stool. Chest X-ray was reported as normal. The result of echocardiography was reported as normal. The pathology of the postoperative resection specimen was not compatible with AA. In the appendix, intact mucosa, lymphoid tissue with the enlarged germinal center, lymphoid hyperplasia, and lymphocyte infiltration without neutrophils in the muscular layer were detected (Figure 2). In the mesoappendix, lymphocyte and histiocyte infiltration was found around the enlarged congested vascular structures (Figure 3).

The patient met MIS-C diagnostic criteria with clinical and laboratory findings. The patient was diagnosed with MIS-C, and treatment was started immediately. A single dose of intravenous immunoglobulin (IVIG) (2 g/kg) was given as treatment. Additionally, methylprednisolone (2 mg/kg/day), aspirin (4 mg/kg/day), and cefotaxime (empirically) were started. The patient's fever decreased to normal at the 12^th^ hour after IVIG treatment. In the follow-up, clinical findings improved, and inflammatory markers regressed. Antibiotherapy was discontinued on the eighth day of hospitalization, as no growth was detected in blood, urine, and stool cultures. The patient was discharged at the end of the ninth day to continue prednol and aspirin therapy. The patient's control examination was normal.

## Discussion

Until recently, it was reported that the clinical course of COVID-19 disease in children was mild. However, it was observed that a systemic disease developed in some children who had COVID-19 in the future, and it was determined that this disease involved many systems and even in some cases caused a systemic reaction similar to Kawasaki Disease. Later, this disease was defined as MIS-C [1,3]. Although MIS-C is rare, it is a serious disease. In the absence of treatment, the need for intensive care may be required, and even it causes fatal complications [4]. 

Clinical manifestations reflect dysfunction, fever, and hyper inflammation that occurred in many organs, including the heart, lungs, kidneys, liver, skin, eyes, brain, and GI tract [3,4]. Cardiac involvement is observed in the majority of cases. Godfred-Cato et al. [5] reported that 41.9% of MIS-C cases were administered vasoactive drugs. No cardiac involvement was observed in this case.

Feldstein et al. [1] reported that the most frequently affected system was the GI system (92%), followed by the cardiovascular (80%), hematological (76%), mucocutaneous (74%), and respiratory (70%) systems. Radia et al. [6] reported that 71% of MIS-C cases presented with GI system findings. Nausea, vomiting, abdominal pain, and diarrhea are common GI symptoms [4,6]. Yock-Corrales et al. [7] reported that COVID-19 or MIS-C is an important factor in the etiology in patients presenting with GI symptoms. In this case, there were symptoms of nausea, vomiting, abdominal pain, and diarrhea, indicating GI system involvement. In addition, among other system involvements, there was hematological system involvement manifested by high D-dimer and fibrinogen.

The appearance and size of the appendix on USG help us in the diagnosis of AA. Martin et al. [8] reported the diameter of the appendix on USG as 7 mm in a case with MIS-C. Jackson et al. [9] reported the diameter of the appendix as 9 mm in their case reports. The appendix diameter of this case on USG was 6.8 mm. Our conclusion from these cases: due to the increased inflammation in MIS-C, inflammation also occurs in the appendix, and accordingly, the size of the appendix increases. This situation can be interpreted in favor of AA. For this reason, it should be considered that there may be an enlargement of the appendix in MIS-C due to inflammation, and MIS-C should be excluded before diagnosing AA. 

Pathological examination of the resection specimen is important after the operation for AA. Jackson et al. [9] in, the pathology examination of their cases, found findings incompatible with AA were in the appendix and small intestine segment. They reported that these findings include inflammation, necrotizing lymphadenitis, and vasculitis [9]. Pathology examination of this case was also found incompatible with AA. In this case, findings including inflammation in the appendix and mesoappendix were determined. Pathological findings in both cases were consistent with a systemic hyperinflammatory disorder. We think that the pathological examination of resection specimens of more MIS-C cases mimicking AA will contribute to the determination of the pathogenesis of MIS-C.

## Conclusions

It should be kept in mind that MIS-C may present with different clinical findings. It should be known that if not treated, it can lead to complications that can result in death. We think that MIS-C should be excluded, especially during this pandemic period, before diagnosing AA in patients with GI symptoms and findings of AA. Thus, unnecessary appendectomy will be prevented. We think that the awareness of all physicians dealing with children, especially pediatricians and pediatric surgeons, on this issue should be increased. We think that publishing such case reports in the literature will both increase awareness about this disease and contribute to the development of preventive and therapeutic strategies.
